# The Analgesia Nociception Index’s Performance During Remimazolam-Based General Anesthesia: A Prospective Observational Study

**DOI:** 10.3390/medicina61040742

**Published:** 2025-04-17

**Authors:** Joohyun Lee, Jung-Min Yi, Young Joo

**Affiliations:** 1 Department of Anesthesiology and Pain Medicine, CHA Ilsan Medical Center, CHA University, Ilsan 13496, Republic of Korea; hightothesky1004@gmail.com; 2 Department of Anesthesiology and Pain Medicine, Asan Medical Center, Seoul 05505, Republic of Korea; jminyi19@gmail.com

**Keywords:** remimazolam, general anesthesia, intraoperative, analgesia nociception index (ANI), nociception, monitoring, tetanic stimulation, equipment

## Abstract

*Background and Objectives*: The Analgesia Nociception Index (ANI), a surrogate marker derived from heart rate variability (HRV) analysis, has been validated for assessing the balance between antinociception and nociception during propofol anesthesia. The ANI continuously monitors this balance, with values above 50 indicating optimal analgesia. By adjusting analgesic administration based on ANI values, anesthesiologists can provide more personalized intraoperative pain control. Remimazolam, a novel benzodiazepine anesthetic lacking intrinsic analgesic properties, exhibits distinct HRV patterns compared to propofol. Considering these differences, the validity of the ANI during remimazolam anesthesia remains uncertain. We evaluated the validity of the ANI by assessing its ability to detect nociceptive stimuli during remimazolam anesthesia. *Materials and Methods*: In total, 28 patients were administered general anesthesia using remimazolam and remifentanil. We evaluated changes in the ANI before and after tetanic stimulation. In addition, we investigated the association between hemodynamic responses during surgical incisions and changes in the ANI. *Results*: Tetanic stimulation resulted in a significant (*p* < 0.001) reduction in the ANI, from 62.0 (interquartile range [IQR] 50.5–76.0) to 44.0 (IQR 37.0–55.5). Of the 13 patients who experienced hemodynamic responses during surgical incision, the ANI significantly decreased from 63.2 ± 13.6 to 36.9 ± 13.8 following noxious surgical stimulation (*p* < 0.001). *Conclusions*: The ANI reflects the dynamic equilibrium between antinociception and nociception during remimazolam-based general anesthesia.

## 1. Introduction

Remimazolam, a recently developed intravenous benzodiazepine sedative, structurally similar to midazolam, has distinct pharmacokinetic characteristics such as rapid onset, ultrashort duration of action, and predictable dose–response relationships [1,2]. These properties render it suitable for use in general anesthesia, whereas midazolam, which has a prolonged context-sensitive half-time, is limited to procedural sedation [3].

Unlike propofol—the most commonly used intravenous agent for general anesthesia due to its favorable pharmacokinetic profile—remimazolam has fewer cardiodepressant effects [4], which is thought to result from differences in its influence on the autonomic nervous system compared to propofol. While this distinction provides an advantage for maintaining hemodynamic stability during general anesthesia with remimazolam, it also raises concerns about whether anesthesia-monitoring devices, such as the Analgesia Nociception Index (ANI), which have been validated for propofol-based anesthesia, remain valid for remimazolam-based anesthesia.

The ANI was designed to predict hemodynamic reactivity associated with inadequate analgesia during general anesthesia [5]. ANI values are derived from the analysis of the high-frequency spectrum of heart rate variability (HRV), reflecting the ratio of parasympathetic to sympathetic nervous system activity. These values range from 0 to 100, with higher values indicating parasympathetic dominance. Theoretically, the ANI provides real-time insight into the balance between analgesia and nociception during general anesthesia, with values > 50 being recommended. Based on the ANI value, anesthesiologists can determine an individualized intraoperative dose of analgesics, enabling more efficient and personalized pain management during general anesthesia.

Many studies conducted on various anesthetic strategies, including propofol and sevoflurane-based general anesthesia, have already demonstrated that ANI values significantly decrease during nociceptive periods—confirming their responsiveness to pain stimuli [6,7]—and that ANI monitoring significantly reduces intraoperative opioid consumption [6], supporting its recommended use in clinical practice.

Recently, Kim et al. [8] demonstrated favorable outcomes with ANI-guided remimazolam-based sedation during the transurethral resection of bladder tumors, suggesting that ANI monitoring may also be useful in remimazolam anesthesia. However, because the impact of remimazolam on autonomic nervous activity and HRV differs from that of propofol [9], its influence on ANI values remains unclear. This discrepancy raises concerns regarding the reliability of ANI measurements in remimazolam-based general anesthesia.

To the best of our knowledge, no studies have directly evaluated whether the ANI remains a valid nociception-analgesia monitoring tool during remimazolam-based general anesthesia—particularly in terms of its responsiveness to nociceptive stimuli and its ability to predict hemodynamic reactivity. Therefore, this study addressed this knowledge gap.

## 2. Materials and Methods

### 2.1. Study Design and Procedure

This prospective observational study included adult patients scheduled for elective robot-assisted gynecological surgery—including total hysterectomy, salpingectomy, myomectomy, and ovarian cystectomy—at Ilsan CHA Hospital between December 2023 and April 2024. Eligible participants were aged between 20 and 79 years and had American Society of Anesthesiologists physical status (ASA PS) I or II. The Institutional Review Board of Ilsan CHA Hospital approved the study protocol (approval number: 2023-04-003-005), and it was registered at ClinicalTrials.gov (NCT06432894). Exclusion criteria comprised autonomic nervous system disorders, arrhythmias, history of neurosurgery, psychiatric disease, epilepsy, pregnancy, neuromuscular disorders associated with spontaneous pain, and current sedative use.

All patients fasted from midnight and withheld medications on the day of surgery. Upon arrival in the operating room, standard monitoring was initiated, including electrocardiography, pulse oximetry, and non-invasive arterial pressure (IntelliVue MX700, Philips America Corporation, Andover, MA, USA). Additionally, patient state index (PSI) (SedLine^®^, Masimo Corporation, Irvine, CA, USA) and ANI (MetroDoloris Medical Systems, Lille, France) monitoring began. General anesthesia was induced with remimazolam at 6 mg/kg/h. After rocuronium administration (0.6–1 mg/kg) to facilitate mask ventilation, the remimazolam infusion rate was titrated to maintain a SedLine^®^ PSI value < 60 during anesthesia maintenance (target range: 1–2 mg/kg/h). Target effect-site concentrations (*Ce*) of remifentanil were adjusted to achieve hemodynamic stability, maintaining blood pressure and heart rate within normal ranges [10]. Neuromuscular blockade assessment was performed using the Train of Four (TOF) ratio (ToFscan^®^, Drager Technologies, Montréal, QC, Canada). Endotracheal intubation was performed when the TOF ratio was <2. Subsequently, patients underwent ventilation in a volume-controlled mode at a respiratory rate of 12 breaths per minute. Tidal volume was adjusted during surgery to maintain the end-tidal carbon dioxide between 35 and 40 mmHg. Finally, the radial artery was cannulated using a FloTrac^®^ transducer (Edwards Lifesciences, Irvine, CA, USA).

**Figure 1:**
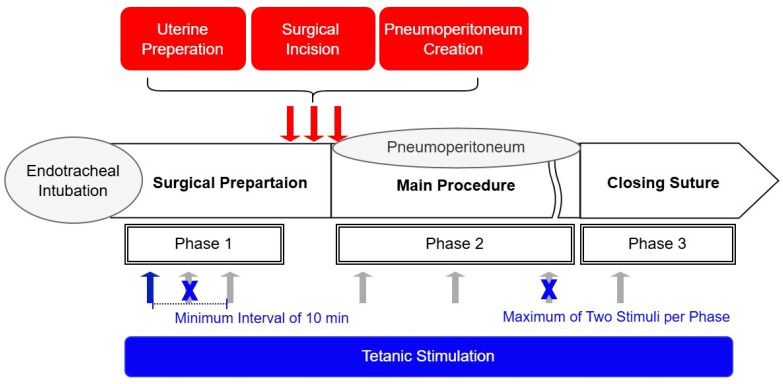
Schematic of study methodology. Tetanic stimulation (60 mA, 50 Hz, 10 s duration) (blue and gray arrows) was applied to patients, and pre- and post-stimulation ANI values were recorded to assess the response of the ANI to noxious stimuli. The *Ce* of remifentanil (antinociception) was titrated based on vital signs in accordance with standard anesthesia practice, allowing for variation in the nociception–antinociception balance within the same patient depending on the surgical context. In patients in whom a change in the nociception–antinociception balance was observed, tetanic stimulation was applied multiple times to the same patient (up to twice per phase, gray arrows). Phases were defined according to the intensity of surgical noxious stimulation (nociception). Hemodynamic changes following surgical noxious stimulation (red arrows) were measured to compare ANI values before and after the stimulation when a hemodynamic response occurred.

To evaluate the ANI’s responsiveness to nociceptive stimuli, tetanic stimulation (60 mA, 50 Hz, 10 s duration) was applied over the ulnar aspect of the wrist during general anesthesia (Figure 1). To minimize potential harm to patients, the *Ce* of remifentanil was not controlled as a study variable during stimulation in this observational study. Instead, *Ce* was adjusted freely to maintain stable vital signs, consistent with routine clinical practice. Stimulation was applied at least 5 min after achieving a pseudo-steady state between blood and brain concentrations of remifentanil. This ensured stable surgical stimulation intensity and patient vital signs, as confirmed by the attending anesthesiologist. Tetanic stimulation sites were categorized based on surgical progress as follows: Phase I, during surgical preparation after endotracheal intubation; Phase II, during surgical pneumoperitoneum; and Phase III, during closure. A response in ANI values following tetanic stimulation was considered statistically independent if the *Ce* changed more than 20% relative to the previous measurement or if the pre-stimulation ANI differed by more than 20% from the prior measurement. Based on these criteria, ANI values before and after tetanic stimulation were collected multiple times per patient. To mitigate the risk of Type I errors, the number of ANI measurements obtained from a single patient was restricted to a maximum of two per phase. For participants receiving more than two stimulations, a minimum interval of 10 min was maintained between applications.

We investigated the association between ANI activity and hemodynamic responses to noxious stimuli during surgery, including uterine preparation, surgical incision, and subsequent pneumoperitoneum creation. To assess the predictive ability of the ANI, we measured the occurrence of hemodynamic changes after these surgical events. Hemodynamic changes were defined as a dichotomous outcome variable, characterized by a > 20% increase in heart rate or systolic blood pressure within 5 min of surgical stimulation.

### 2.2. Data Acquisition

During surgery, we collected data regarding ANI values, vital signs, remifentanil *Ce*, the remimazolam infusion rate, and ventilator settings. Real-time data, excluding ANI values, were retrieved using an RS232C cable connected to the Vital Recorder and downloaded directly to personal computers for subsequent analysis [11]. ANI data were acquired from the Root^®^ device (Masimo Corporation, Irvine, CA, USA) using a separate RS232C cable after surgery and downloaded to the same computer.

### 2.3. Statistical Analyses

Sample size determination was based on prior studies evaluating the ANI in patients undergoing general anesthesia [12]. Considering a dropout rate of 20%, 30 participants were recruited. Statistical analyses were performed using SigmaStat 4.0 (Graffiti, Palo Alto, CA, USA) and GraphPad Prism (ver. 10.2.3, GraphPad Software, Boston, MA, USA). Continuous variables were assessed using the *t*-test or the rank-sum test.

The ANI value measured immediately before noxious stimuli in each volunteer was compared with the minimum value recorded within 2 min after tetanic or surgical stimulation [12]. Receiver operating characteristic (ROC) curves were used to determine optimal cut-off values for the ANI that maximized sensitivity and specificity in detecting hemodynamic responses. Cut-off values were determined to maximize the combined sensitivity and specificity of the test. Continuous variables are expressed as means ± standard deviations for normally distributed data, whereas non-normally distributed data are presented as medians (25–75%). Categorical variables are presented as numbers and percentages. *p*-values < 0.05 were considered statistically significant.

## 3. Results

Initially, 30 patients who underwent surgery were included. None met the predefined exclusion criteria; however, two patients were excluded due to technical issues with intraoperative ANI data recordings. Patient characteristics are summarized in Table 1.

We collected 78 pairs of ANI data before and after tetanic stimulation. After data exclusion, 61 pairs (21, 31, and 9 from Phases I, II, and III, respectively) were analyzed. Data were excluded if remifentanil *Ce* was adjusted within 2 min of tetanic stimulation or if ANI values were missing for >50% of the recorded 2 min period after stimulation.

**Figure 2:**
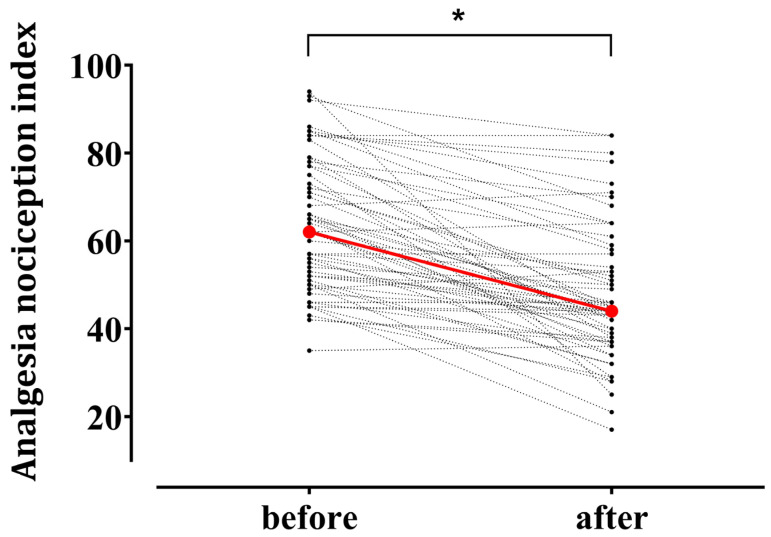
Individual changes in the Analgesia Nociception Index (ANI) after tetanic stimulation in patients under general anesthesia using remimazolam (n = 61). Red circles represent median values at the baseline 62.0 [50.5–76.0] (median [interquartile range]) and after tetanic stimulation 44.0 [37.0–55.5]. * *p* < 0.05.

Figure 2 illustrates individual changes in the ANI after tetanic stimulation. The median ANI value exhibited a significant decrease from the baseline 62.0 (50.5–76.0) to 44.0 (37.0–55.5) after noxious stimulation (*p* < 0.001). The mean remifentanil *Ce* during tetanic stimulation was 7.8 (4.5–10.0) ng/mL.

**Figure 3:**
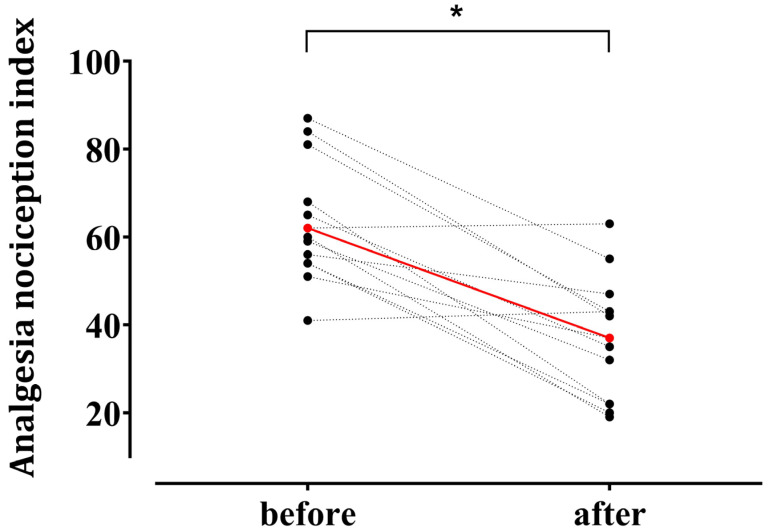
Individual changes in the Analgesia Nociception Index (ANI) after surgical noxious stimulation in patients who exhibited hemodynamic responses (n = 13). Among 28 enrolled patients, 13 demonstrated a hemodynamic response—defined as an increase in heart rate or mean blood pressure ≥ 20% from the baseline—within 5 min after a surgical noxious stimulus (uterine preparation, skin incision, or pneumoperitoneum creation). Red circles indicate mean values at the baseline (63.2 ± 13.6) and after stimulation (36.9 ± 13.8) (mean ± standard deviation). * *p* < 0.05.

Hemodynamic responses to surgical incision occurred in 13 patients. ANI values decreased from 63.2 ± 13.6 before incision to 36.9 ± 13.8 after surgical noxious stimulation (*p* < 0.001) (Figure 3).

**Figure 4:**
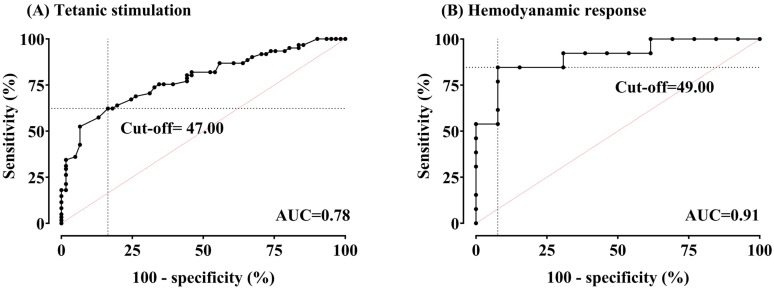
(**A**,**B**) Receiver-operating characteristic curves for the Analgesia Nociception Index (ANI) in detecting tetanic stimulation (pain) and hemodynamic response (HemoR). Areas under the curve (AUCs) were 0.78 (95% confidence interval (CI) 0.70–0.86, *p* < 0.0001) for pain and 0.91 (95% CI 0.79–1.00, *p* < 0.0001) for HemoR. The optimal cut-off value for pain was 47.00, with a sensitivity of 62.3% and specificity of 83.61%. For HemoR detection, the optimal cut-off value was 49.00, with a sensitivity of 84.62% and specificity of 92.31%.

Figure 4 presents areas under the ROC curves and optimal cut-off values for detecting noxious stimulation and hemodynamic change using the ANI.

## 4. Discussion

Remimazolam is a novel drug with limited data beyond its basic pharmacokinetic and pharmacodynamic properties. Therefore, determining the applicability of standard anesthetic monitoring devices, which are crucial for the individualized control of intraoperative drug concentrations, in patients undergoing remimazolam-based general anesthesia is essential. Although numerous studies have reported the characteristics of remimazolam, the focus has primarily been on its effects on electroencephalogram (EEG)-derived hypnotic indices, such as the Bispectral Index and PSI, likely reflecting its anesthetic properties [2].

Intraoperative pain perception remains difficult to assess in patients under general anesthesia due to the lack of verbal communication. Traditionally, pain during surgery is evaluated based on changes in vital signs. Because these changes are nonspecific, conventional methods cannot reliably indicate whether surgical stimuli are perceived as pain. Anesthesiologists administer analgesics up to the maximum allowable dose, maintaining vital signs above a threshold based on the anticipated intensity of the surgical stimuli, to preemptively block potential pain. This approach, based on standardized protocols for opioid infusion rates during surgery, does not allow for individualized titration of analgesic doses according to each patient’s specific needs. Over the past decade, methods for directly monitoring the response of the autonomic nervous system to surgical stimuli in real-time have been developed [5,7], with one such method, the ANI, being commercialized to assess the patient’s parasympathetic activity during surgery. Using this device, anesthesiologists can continuously monitor the level of pain a patient experiences in response to surgical stimuli, ensuring that each patient receives an optimal dose of analgesics based on their individual physiological response.

The ANI uses HRV analysis from the electrocardiogram signal to estimate the balance between nociception and analgesia [5]. Considering the limited understanding of the analgesic properties of remimazolam, its administration should not alter patient pain perception. Consequently, irrespective of remimazolam use, the ANI should reflect this balance during general anesthesia. However, a previous study demonstrated potential differences in HRV characteristics between remimazolam and propofol [9]. This previous study examined how the relative power of low-frequency power (LF), representing sympathetic nervous system activity, and high-frequency power (HF), representing parasympathetic nervous system activity, varies before and after inducing anesthesia with either propofol or remimazolam, as assessed by the power spectral density of HRV. In patients anesthetized with propofol, an increase in the LF ratio and a decrease in the HF ratio were observed compared to the pre-anesthesia state. In contrast, no significant changes in either the LF or HF ratios were observed in patients anesthetized with remimazolam. Given that the ANI reflects relative parasympathetic activity based on signals from the HF region of HRV, the observed differences in HRV signal analysis induced by different anesthetic agents emphasize the need to validate the efficacy of the ANI, which has been validated in propofol-based general anesthesia, in remimazolam-based general anesthesia.

We investigated the ANI’s effectiveness during general anesthesia using remimazolam. We observed a significant decrease in ANI values upon tetanic stimulation application to patients under balanced surgical nociception and analgesia. This finding suggests that the ANI can discriminate additional noxious input, even with remimazolam-based anesthesia. Some studies assessing the ANI’s functionality during anesthesia define “pain” as hemodynamic responses triggered by noxious stimuli rather than noxious stimuli themselves; ROC curves evaluate the ability of the ANI to detect these hemodynamic responses. This approach validates the ability of the ANI to detect physiological changes associated with pain. We confirmed these findings with a high area under the ROC curve of 0.91, supporting the effectiveness of the ANI during remimazolam-based anesthesia.

Consistent with our findings, prior studies using propofol or sevoflurane for general anesthesia demonstrated that the ANI could detect pain stimuli, such as intubation, surgical incision, and tetanic stimulation. These studies documented significant changes in ANI values after such events [12,13,14,15,16]. Although detailed numerical variations exist, prior studies consistently established the effectiveness of the ANI.

Previous studies investigating the area under the curve for detecting noxious stimulation in propofol-anesthetized patients reported values ranging from 0.88–0.99 [7]. Our findings demonstrate similar discriminative ability, suggesting that the ANI remains a reliable discriminator of noxious stimulation under remimazolam. However, the cut-off values for tetanic stimulation and surgical incision in our study (47 and 49, respectively) differed from the previously reported range of 38–63. These variations likely arose from methodological differences and patient population characteristics. Several studies have demonstrated varying cut-off ANI values depending on the surgical procedure and patient population. For example, a cut-off of 38 was identified for detecting tetanic stimulation during radical prostatectomy with laryngeal mask airway insertion [13]. Conversely, sedated patients undergoing suspension laparoscopy required a value of 55 to predict hemodynamic response, and a value of 63 was associated with tibial osteotomy during total knee replacement under general anesthesia [17,18]. Although an ANI value ≥ 50 is recommended during monitoring, the titration of analgesic administration based on individual patient trends (rather than relying solely on absolute values) may be more beneficial for pain management.

The optimal concentration of analgesics during surgery depends on the surgical phase and the intensity of nociceptive stimuli. Anesthesiologists must continually adapt analgesic infusion rates based on real-time assessments. This is particularly challenging for anesthetized patients who cannot communicate their pain perception. Although the ANI has been investigated as a potential pain assessment tool, its validity in this specific clinical scenario remains uncertain.

Prior studies have primarily investigated the ability of the ANI to detect externally applied noxious stimuli compared to the non-nociceptive period before surgical incision [13,15,17,19]. Several others have examined correlations between ANI values and analgesic concentrations (remifentanil or fentanyl) in the absence of surgical noxious stimulation [12,16,20]. Although results have consistently shown that the ANI can distinguish nociceptive from non-nociceptive states, findings regarding its correlation with analgesic concentrations have been inconsistent—likely due to individual differences in nociceptive sensitivity and responsiveness to opioids.

Given these differences, applying a fixed remifentanil dosage during surgery may lead to under- or over-treatment, potentially compromising patient safety. Therefore, rather than standardizing remifentanil concentrations, we designed this study to maintain a dynamic balance between nociception and analgesia based on each patient’s real-time condition. Accordingly, unlike previous studies that used fixed tetanic stimulation timing and standardized remifentanil concentrations, we implemented a variable stimulation regimen (with only 34% of all pairs devoid of surgical stimuli) and maintained individualized remifentanil dosing. Notably, we assessed if ANI values decreased with additional nociceptive stimuli while ensuring a balance between nociception and analgesia during anesthesia, mirroring real-world clinical scenarios where anesthesiologists require real-time ANI guidance. Therefore, unlike previous studies conducted under controlled experimental conditions with fixed stimulation and standardized medication regimens, our research design strengthens the validity of the ANI by reflecting real-world clinical scenarios.

Our study had several limitations. First, the generalizability of our findings may be limited due to a gender imbalance in enrolled participants. Because this study was conducted in an operating room primarily dedicated to gynecological surgeries, where ANI monitoring and vital sign recording routinely occur, all included participants were female. Although our findings demonstrate the efficacy of the ANI during general anesthesia using remifentanil in the female population, further studies are necessary to investigate the applicability of the ANI in other patient groups receiving remifentanil. Second, although older patients were included in this study, the mean age was relatively low (42 ± 7.5 years). Given that autonomic nervous system function may change with age, the validity of ANI monitoring in older populations should be explored in future research. Third, although 61 intraoperative data points were analyzed, relatively few patients were enrolled (n = 28). Although this sampling strategy enabled the evaluation of the ANI’s performance across various nociceptive states, future studies with larger patient populations are warranted to confirm the generalizability of our findings. Fourth, potential bias may arise from the variable number of samples collected per patient. These samples were independently analyzed without consideration of individual patient characteristics. However, although multiple samples were obtained from each participant, they were collected at distinct surgical stages or under varying remifentanil concentrations. Consequently, these samples can be better interpreted as reflecting a spectrum of surgical conditions rather than replicating individual patient responses. Finally, this study did not directly assess the pharmacodynamic interaction between remimazolam and remifentanil. Because remifentanil dosing was individualized based on clinical judgment, the potential modulatory effects between these two agents on ANI values could not be fully elucidated. Further studies using controlled remifentanil dosing protocols may help clarify the nature of their interaction.

Despite these limitations, our study is the first to validate the ability of the ANI to discriminate noxious stimulation in patients under general anesthesia using remimazolam. These findings provide valuable preliminary insights for ANI use in anesthesia settings using remifentanil, warranting further studies. 

## 5. Conclusions

In this context, the importance of this study lies in its provision of a fundamental basis for the potential use of the ANI in enabling individualized opioid titration, while maintaining more stable vital signs during general anesthesia with remimazolam.

## Figures and Tables

**Table 1:** Characteristics of the study population.

	Patients (n = 28)
Male/Female	0/28
Age, yr	42 ± 7.5
Height, cm	161 ± 5
Weight, kg	61 ± 8
ASA PS 1/2	6/22
Duration of surgery, min	105 ± 41
Duration of general anesthesia, min	129 ± 42
Mean infusion rate during anesthesia ^a^	
Remimazolam, mg/kg/h	1.14 [1.10–1.18]
Remifentanil, mcg/kg/min	0.44 ± 0.15
Number of patients receiving coadministration, n (mean dose ^b^)	
Atropine	1 (0.2 mg)
Ephedrine	3 (5 mg)
Phenylephrine	1 (50 mcg)
Nicardipine	2 (600 mcg)

Data are expressed as the mean ± SD or median [interquartile range] as appropriate. ^a^ The mean infusion rate was calculated as follows: mean infusion rate = total dose administered during anesthesia/(body weight × infusion duration). ^b^ The mean dose for patients receiving each drug. ASA PS, American Society of Anesthesiologists physical status.

## Data Availability

Original contributions presented in this study are included in the article. Further inquiries can be directed to the corresponding author.
